# Building research capacity in Botswana: a randomized trial comparing training methodologies in the Botswana ethics training initiative

**DOI:** 10.1186/1472-6920-13-14

**Published:** 2013-02-01

**Authors:** Francis H Barchi, Megan Kasimatis-Singleton, Mary Kasule, Pilate Khulumani, Jon F Merz

**Affiliations:** 1 School of Social Work, Rutgers, The State University of New Jersey, 536 George Street, New Brunswick, NJ, 08901-1167, USA; 2 Department of Medical Ethics and Health Policy, Perelman School of Medicine, University of Pennsylvania, 3401 Market Street, Suite 320, Philadelphia, PA, 19107-3319, USA; 3 Council on Health Research for Development, 1-5 Route des Morillons, P.O. Box 2100, Geneva, 1211, Switzerland; 4 Health Research Unit, Ministry of Health, Republic of Botswana, Private Bag 0038, Gaborone, Botswana

## Abstract

**Background:**

Little empirical data are available on the extent to which capacity-building programs in research ethics prepare trainees to apply ethical reasoning skills to the design, conduct, or review of research. A randomized controlled trial was conducted in Botswana in 2010 to assess the effectiveness of a case-based intervention using email to augment in-person seminars.

**Methods:**

University faculty and current and prospective IRB/REC members took part in a semester-long training program in research ethics. Participants attended two 2-day seminars and were assigned at random to one of two on-line arms of the trial. Participants in both arms completed on-line international modules from the Collaborative Institutional Training Initiative. Between seminars, intervention-arm participants were also emailed a weekly case to analyze in response to set questions; responses and individualized faculty feedback were exchanged via email. Tests assessing ethics knowledge were administered at the start of each seminar. The post-test included an additional section in which participants were asked to identify the ethical issues highlighted in five case studies from a list of multiple-choice responses. Results were analyzed using regression and ANOVA.

**Results:**

Of the 71 participants (36 control, 35 intervention) enrolled at the first seminar, 41 (57.7%) attended the second seminar (19 control, 22 intervention). In the intervention arm, 19 (54.3%) participants fully completed and 8 (22.9%) partially completed all six weekly cases. The mean score was higher on the post-test (30.3/40) than on the pre-test (28.0/40), and individual post- and pre-test scores were highly correlated (r = 0.65, p < 0.0001). Group assignment alone did not have an effect on test scores (p > 0.84), but intervention-arm subjects who completed all assigned cases answered an average of 3.2 more questions correctly on the post-test than others, controlling for pre-test scores (p = 0.003).

**Conclusions:**

Completion of the case-based intervention improved respondents’ test scores, with those who completed all six email cases scoring roughly 10% better than those who failed to complete this task and those in the control arm. There was only suggestive evidence that intensive case work improved ethical issue identification, although there was limited ability to assess this outcome due to a high drop-out rate.

## Background

Many countries in the global south are hard-pressed to identify in-country personnel with adequate training in human subjects research ethics to participate as investigators, research staff, or members of ethics review bodies. International partners have attempted to address this need by incorporating research ethics short courses and workshops into their capacity-building programs or by supporting host-country initiatives to implement training efforts of their own. While such programs generally share a common goal – that of building competency in human subjects’ protections – they can vary significantly in instructional approach and format. Some programs focus on formal guidelines, general ethical principles and historically noteworthy cases of research abuse; these programs provide researchers with information needed to meet regulatory requirements but do not necessarily prepare them to apply ethical reasoning skills to the design and conduct of research [1]. Such programs, which emphasize compliance with national regulations and international guidelines, are easier to develop than case-based instruction, can be taught over relatively short periods of time, and can be readily compared to existing regulations to ensure that all required elements have been covered [2].

Studies, however, have suggested that researchers are more likely to formulate their concepts of scientific integrity and responsibilities towards others from climate, institutional social contexts, and ethical norms than from formal guidelines [3,4]. Research ethics education programs that combine lectures with inter-collegial discussion of case-based ethical situations may be more effective in fostering recognition of ethical issues, capacity for moral reasoning, intentionality, and the ability for action [1,5,6].

International research ethics training programs also vary significantly in format, including on-site, in-person short courses and workshops [6-8] wholly on-line distance education courses [9-14] and hybrid programs combining face-to-face instruction with supplementary web-based materials. In addition, a number of international collaborations have produced ethics training curricula in modular form which are available in print, online, or as CD-ROMs and which can be tailored to meet the training needs, resources, and time constraints of individual programs or institutions [6,15,16]. Face-to-face workshops and short-courses can be valuable given the opportunity they provide for discussion, debate, and exchange of ideas, but are resource-intensive and rely on the availability of participants and faculty with dedicated time to commit to such purposes [6]. On-line programs, which offer maximal flexibility in use and require relatively few resources once established, have the added advantage of accessibility for large numbers of people with different schedules and learning speeds. A 2009 quantitative meta-analysis of ethics program evaluation efforts, however, found that ethics courses which emphasized student engagement through highly interactive learning and practice activities promoted instructional effectiveness over those which relied more on self-directed learning [17]. Other scholars, while they debate the extent to which Internet-based learning can contribute to skill development in ethical-decision making, acknowledge that online courses that require interaction with faculty or other students and which ask trainees to tackle ethical problem-solving in writing, can foster ethical reasoning skills [18]. A recent randomized controlled trial comparing on-line and on-site training programs in biostatistics and research ethics, for example, found marked and similar improvements among volunteer scientists, suggesting that on-line instruction may offer a cost-effective, scalable alternative to more resource-intensive face-to-face instructional programs [19]. Evaluators of hybrid programs combining face-to-face interaction with on-line learning emphasize the importance of the face-to-face component to establish collaborative atmospheres in which individuals can safely engage in dialog over controversial ethical issues [20].

### Research ethics in Botswana

With one of the highest rates of HIV in the world, a stable democracy, and a national commitment to public health, Botswana has attracted global attention over the past three decades as a hub for behavioural, epidemiological, and clinical research related to HIV-AIDS and other co-morbid conditions. A number of foreign academic research universities and NGOs have established study centers in Gaborone, the capital city, in order to support on-going research activities. Post-graduate opportunities in the country’s tertiary educational institutions have also expanded during the past decade, resulting in a growing national research portfolio. The rapid increase in the volume and complexity of research activity in the country has necessitated the strengthening of the national research regulatory system and has generated a demand for a trained national workforce able to conduct ethics reviews or serve as investigators and staff at all levels of the research enterprise.

Ethics review of all health and health-related research in Botswana is currently conducted by a government-appointed national research ethics body, the Health Research and Development Committee (HRDC), which functions as an Institutional Review Board/Ethics Review Committee (IRB/REC) with administrative support from the Health Research Unit at the Ministry of Health (HRU). The HRDC increasingly relies on the IRB/REC at the University of Botswana (UB) for research conducted by its faculty and students, but retains authority for final review and approval of all international protocols. A number of IRBs have been established at the local level in hospitals and academic centers, but these, like the HRDC and the IRB/REC at UB, suffer from a paucity of staff and committee members with knowledge and skills in research ethics. Despite concerted efforts in ethics training by the Ministry of Health (MoH) and UB, supported in part by various international agencies such as the Fogarty International Center at the National Institutes of Health, the Wellcome Trust, and the European and Developing Countries Clinical Trials Partnership (EDCTP), the number of individuals in-country with adequate training in research ethics to meet growing demand remains quite small [21-23]. The same limited pool of individuals is often pressed into service on multiple projects or ethics committees. Local entities that have sponsored or endorsed their staff members’ participation in formal academic ethics training programs often lose them on completion of their training to more lucrative jobs with regional or international organizations.

Limited empirical data on the effectiveness of various instructional approaches in teaching research ethics are available to guide Botswana institutions as they expand their training efforts. The traditional practice among the Batswana people to discuss issues of importance in familial and communal settings would suggest that face-to-face small-group problem-solving would be highly effective. Wholly on-line instruction, despite its advantages as a low-cost, scalable, flexible medium, requires reliable Internet access, a rarity in Botswana where Internet service, even within government and academic centers, is sporadic. Use of email as a communication tool, however, is fairly common, particularly among Batswana professionals, although access to computers with connectivity is frequently limited to the workplace or Internet cafes.

In 2010, with support from EDCTP, the MoH launched a multi-year endeavour to create a country-wide system of ethics committees/community advisory boards in response to the growing demand for ethics review of student-initiated research in academic institutions situated outside of the capital city as well as the interest of the research community in developing new study sites in many of these same locations. Seven communities were the foci of this proposed system, selected because they were sites of satellite campuses of the Institute of Health Sciences (IHS), a tertiary educational institution responsible for the training of nurses and affiliated health professionals, as well as government-run District Health Teams (DHTs) which serve the semi-urban settlements of Batswana. The MOH proposed to create local ethics committees that would jointly serve as IRB/RECs of record for student-initiated research and as community advisory boards to the HRDC on international research situated within their communities.

In implementing this ethics capacity-building initiative, the Ministry faced a number of challenges relating to course content, appropriate teaching methodologies, and faculty. A grant from the Penn Center for AIDS Research enabled study authors FHB and JFM to work with professional ethics staff at the MOH and UB to offer training to researchers, and ethics committee members from across Botswana. The resultant training program was also used as an opportunity to study the effectiveness of an Internet based learning environment in this population.

## Methods

A randomized controlled trial was conducted in Gaborone, Botswana in 2010 to assess the effectiveness of a case-based personalized intervention using email to augment in-person ethics training seminars. Participants enrolled in a semester-long course in research ethics that included two 2-day in-person seminars separated by a six-week long on-line component. Participants were assigned at random to one of two arms for the on-line portion of the course. Control arm participants were to complete two on-line international research ethics training modules available on-line from the Collaborative Institutional Training Initiative (CITI) [24]. Participants in the intervention arm were assigned the same CITI modules to complete and in addition were asked to review and comment on one case sent to them via email weekly during the six-week interim. Course faculty (FHB, MKS) reviewed respondents’ weekly case analyses and returned individualized comments to them each week. All study participants were tested at the start of the training program and again at three months after both arms had completed the CITI modules and the intervention arm had completed the case analyses and email discussions with the study team. The post-test was scheduled in advance of the final seminar in order to assess the effectiveness of the intervention email case exchange rather than the overall training program itself.

**Figure 1:**
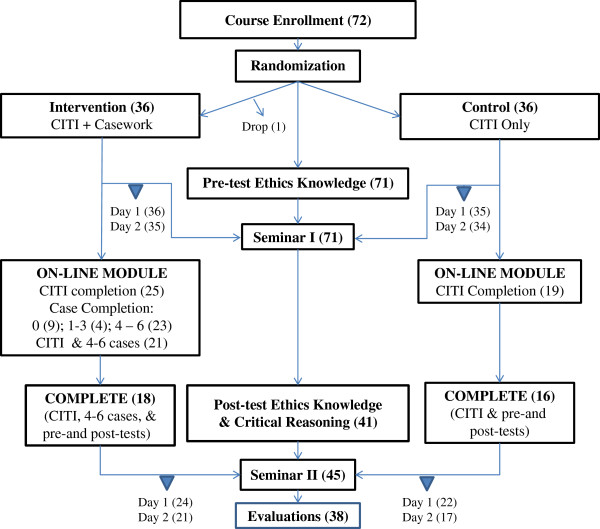
Study design.

The study, illustrated in Figure 1, was designed to explore these hypotheses:

● 1. Ethics knowledge would improve for all trainees in Botswana who participated in on-site seminars in human subjects’ research ethics.

● 2. Greater gains would be made by those trainees who also completed two on-line international research ethics training modules developed by the Collaborative Institutional Training Initiative (CITI).

● 3. Trainees who independently completed a case analysis with personalized faculty feedback by email each week for six weeks would, on examination, demonstrate a greater ability to correctly identify ethical issues than their counterparts who had not.

The project was developed in consultation with the HRU staff at the Botswana MOH and the Office of Research Development (ORD) at UB. It underwent ethics review and was approved by the HRDC in Botswana and the IRB/RECs at UB and the University of Pennsylvania. Participants documented their consent by signature prior to randomization and the start of the first seminar.

The training curricula was developed by study personnel at the University of Pennsylvania Center for Bioethics (FHB and JFM) with input from staff at the HRU (MK and PK) and ORD. Seminar topics detailed below were selected to cover major aspects of human subjects’ protections as well as provide instruction and opportunities for discussion of the values, principles, and regulatory systems that guide research in Botswana. All participants were given loose-leaf binders containing course materials as well as copies of the Declaration of Helsinki, the Belmont Report, the Council of International Organizations of Medical Science (CIOMS) International Ethical Guidelines for Biomedical Research Involving Human Subjects, and the Common Rule [24-28]. Case studies and mock IRB/REC reviews were adapted from existing studies or created de novo for the course. Seminars took place at sites in Gaborone and were led by Penn course faculty (FHB and JFM) and Batswana lecturers drawn from the HRU and UB. All cases and IRB/REC review materials were reviewed in advance with the Batswana faculty to identify key topic areas for discussion during the small-group segments of the seminars. All lectures, discussions, and written materials were in English, the official language of Botswana and the standard language used in educational settings.

Topics for the two face-to-face seminars were as follows:

Seminar 1

● Value-driven research

● Botswana research goals and values

● Major codes and regulations that guide research in Botswana: Declaration of Helsinki, CIOMS, ICH: Guidelines for Good Clinical Practice

● The regulatory ethics structure in Botswana

● Informed consent: What is it? What is required? What do we know about it? What is the IRB’s role?

● Whose consent?: Autonomous subjects, subjects with impaired autonomy, children, vulnerabilitySample selection, recruitment and retention

● Small-group case work

● Consent drafting exercise

Seminar 2

● An ethics model for international collaborative research

● Small-group case work

● Small-group Mock IRB exercises

The CITI International Research Ethics Platform assigned to both trial arms is a free, public-access, on-line program designed for researchers, research staff and research ethics committee members involved in international research [24]. The platform offers two tracks, one which targets international investigators and one which targets non-US investigators who are involved in US Federally-funded international research. Both tracks provide an historical background on research involving human subjects and the evolution of research ethics as well as materials on the research review process, informed consent, international studies, and key ethical guidance documents.

Pre- and post-tests were adapted from materials developed by Family Health International (FHI) for use in its Research Ethics Training Curriculum (RETC) [29]. An additional section developed by FHB and JFM for use in the post-test included five cases with corresponding multiple-choice statements describing potential ethical challenges. Test results were analyzed using regression and ANOVA.

The majority of cases used in the two seminars as well as the intervention arm on-line component were written by FHB in consultation with other members of the study team. Despite the availability of several high-quality casebooks focused on international research ethics [30,31], we felt that participant learning would be enhanced if ethical challenges common to human subjects research everywhere were presented in settings and social groups that reflected the lived experience in Botswana. Participants may find it easier to focus on the ethical dilemmas embedded within situations to which they can relate rather than those that seem unlikely to occur in their communities. In addition, particular social and community norms may themselves introduce an ethical dilemma that would not be seen as such to others unfamiliar with the culture. For example, one of the new cases developed for this program described a study in which a group therapy model was proposed to elicit conversation between mothers and daughters about adolescent sexual behaviors. While the use in any educational setting of such a case would be intended to stimulate discussions about confidentiality and consent, the protocol raised additional ethical as well as practical challenges in Botswana, where discussions and confidences about sexual behaviors traditionally do not take place between mother and daughter but rather between a young woman and another older female relative. Using local examples may enhance learners’ abilities to identify and address ethical dilemmas more effectively than cases in which the problems as well as the steps available for their resolution may not be seen as relevant.

All participants who attended the final day of the seminar series were asked to complete an anonymous survey evaluating various aspects of the program. Certificates of completion were issued to those participants in the control arm who had attended both seminars, completed the assigned CITI modules and sat for both the pre- and post-tests. Trainees assigned to the intervention arm received certificates if they had completed these tasks as well as at least four of the six assigned on-line case analyses.

## Results

Seventy two individuals registered at the first seminar in September 2010 and signed a consent form for participation following a presentation on the research aspects of the training program. One subject withdrew from the study after randomization. The remaining 71 participants included faculty and staff from UB (20) and IHS (9), prospective IRB/REC community members (8), members and staff of the HRDC (9), other Botswana government staff (3), staff employed by US academic research institutions with established programs in Botswana (12), research staff employed by international NGOs (8), and graduate students (2).

The distribution of participants at various stages of the training program is illustrated in Figure 1. Of the 71 individuals who attended the first seminar, 43 (60.6%) completed the CITI training program, and 41 (57.7%) returned for the second training session in December 2010. Those trainees who attended the final seminar were the more motivated participants, with 35 of the 41 (85.4%) completing the CITI training. Of the 35 trainees assigned to the intervention arm, 19 (54.3%) fully completed and eight (22.9%) partially completed the six weekly email cases. Twenty-two of the intervention arm participants (62.8%) returned for the second training session.

Participants were given the FHI 40-item test at the start of each 2-day seminar. As we hypothesized, participant knowledge about ethics improved over the course of the semester, when measured as gains in the number of correct answers to a battery of multiple-choice questions in the post-test compared to pre-test scores on the same battery at the start of the course. Out of 40 questions, participants answered a mean of 28.0 correctly on the pre-test (Range 12–38; SD = 4.9), and 30.3 correctly on the post-test (Range 22–39; SD = 4.4); post-test score was strongly correlated with pre-test score (r = 0.65, p < 0.0001). Group assignment itself did not have a main effect on test scores (p = 0.8), and CITI training appeared to have only a weak effect (p = 0.1). However, we found that subjects who took part in the on-line intervention and provided weekly answers to at least some of the questions did substantially better on the post-test (p < 0.01), and those who analyzed all six email cases answered an average 3.2 more questions correctly on the post-test than others (p = 0.003).

To assess whether our intervention improved subjects’ post-test case-based issue identification, we examined sensitivity – respondents’ ability to detect real issues –and specificity – their erroneous identification of nonissues (or false positive rate), across the 5 test cases. There were a total of 15 “correct” and 14 “incorrect” multiple choice answers for the cases. The 41 post-test subjects on average identified 9.3 (0.62%, range 3–14) correct issues, and 4.1 (0.29%, range 1–9) incorrect issues, corresponding to an average sensitivity of .62 and specificity of .71. Both pre-test (t = 3.71, p = 0.001) and post-test scores (t = 3.91, p < 0.001) were related to higher sensitivity, but had no effect on specificity. Group assignment had no effect on sensitivity or specificity (minimum p = .60), but the subgroup of 19 who completed all 6 email cases had a higher specificity, with an average of just over one fewer false positive nonissues chosen (p = 0.051). CITI training had no effect on sensitivity or specificity (minimum p = 0.92).

## Discussion

In-person seminars proved to be a popular format for our trainees. The use of conference facilities situated away from the workplace seemed to encourage participants to remain for the full-day programs they attended, although the off-site locations may have also contributed to the overall high dropout rate. Retention was high among faculty from the IHS, which supported institutional attendees from each of its seven locations; only one IHS trainee did not attend both seminars. Despite a sizeable pre-registration by UB faculty, turnout and retention at the seminars was small due to the competing demands of an on-campus strike and last-minute changes in grading schedules. While the research component of our effort precluded mandatory participation, consideration should be given in future training programs to the merits of using certification requirements or academic/professional credits to achieve and maintain target levels of participation.

Participants had to be on-line in order to complete the CITI modules and there had been general concern among the study authors at the outset that limited or sporadic Internet access would discourage participants from completing this task. Those who were assigned to the on-line case analyses had the option to work on these off-line, attaching them to an email at times when the Internet was available to them. As we had predicted, participants did complain of recurrent difficulty in accessing the Internet. Of the 38 participants who completed the evaluation, 18 had computers at work, one third of them without Internet access; 20 respondents had computers at home, only nine of which had Internet access. In general, Internet access was characterized as “sketchy” or “not good”. This would suggest that developers of future ethics training programs should determine Internet accessibility prior to opting for more interactive formats such as discussion boards, blogs, and chat rooms.

In general, the use of cases, both in small-group discussions in the seminars, and as assignments in the on-line module, were felt by participants to be an excellent skill-building exercise and the most interesting aspect of the course. Providing personalized feedback on case analyses to each participant proved extremely time-consuming for course faculty, requiring no less than 20 individualized emails for each of the six cases. A number of participants engaged with faculty in multiple email discussions about particular cases and guidance documents; while this email case exchange was highly desirable from a pedagogical perspective, it would have quickly become an unmanageable task for faculty had more participants availed themselves of the opportunity to exchange ideas in this manner.

## Conclusions

We found that completion of our case-based educational intervention using email with distant faculty and personalized feedback improved respondents’ post-test scores, with those who completed all six email cases doing roughly 10% better than those who were in the control arm or those who did not spend the effort to complete the case-based task, controlling for their initial pre-test scores. We hoped this intensive exercise in issue identification would improve participants’ abilities to accurately identify ethical issues in the post-test, and we found suggestive evidence that this was effective in reducing misidentification of nonissues. Due to 42% dropout from the second training session, we had limited ability to assess this outcome.

While this study showed some positive results in the use of case-based email discussions to promote ethics learning, such interaction is extremely time-consuming for both faculty and participants. The benefits of using email case exchanges for research ethics training as stand-alone curricula or to enhance face-to-face seminars should be carefully considered before committing the resources required to do so.

Similar to other research efforts linked to ethics training programs, this study provided no mechanism for on-going evaluation of ethics knowledge retention over time, or for monitoring of how such knowledge would be used in future practice as participants engage in research-related activities involving human subjects. Absent such mechanisms, educators will remain hard-pressed to assess accurately the lasting impact of ethics training programs on the research environment.

## Competing interests

The authors declare that they have no competing interests.

## Authors’ contributions

FHB developed the project design, workshop curriculum and cases, served as faculty in the seminars and the on-line case-based intervention, supervised data collection, and wrote draft manuscripts. JFM reviewed and contributed to project design curriculum and cases, served as faculty in the seminars, designed and implemented data analysis plan, and reviewed and edited draft manuscripts. MKS served as faculty for the on-line case-based intervention and reviewed and edited draft manuscripts. MK and PK contributed to the conceptualization of the project, identified and recruited study participants, organized in-country logistics, and served as faculty in the seminars. All authors read and approved the final manuscript.

## Pre-publication history

The pre-publication history for this paper can be accessed here:

http://www.biomedcentral.com/1472-6920/13/14/prepub
